# An unusual case of epidermolysis bullosa complicated by persistent oligoarticular juvenile idiopathic arthritis; lessons to be learned

**DOI:** 10.1186/1546-0096-9-13

**Published:** 2011-06-27

**Authors:** Despoina Maritsi, Anna E Martinez, Jemima E Mellerio, Despina Eleftheriou, Clarissa A Pilkington

**Affiliations:** 1 Rheumatology Department, Great Ormond Street Hospital NHS Trust, London, UK; 2 Dermatology Department, Great Ormond Street Hospital NHS Trust, London, UK

## Abstract

Recessive Dystrophic Epidermolysis Bullosa (RDEB) is a rare and severe hereditary skin disease. Oligoarticular Juvenile Idiopathic Arthritis (JIA) although infrequent in the general paediatric population, is the most frequent type of autoimmune joint disease in children. While different in aetiology, both diseases are characterized by gradual deterioration in mobility and function. We report a female patient, diagnosed with RDEB at birth, who presented with inflammatory bowel disease (IBD) at the age of four years, and subsequently developed oligoarticular JIA at seven years of age, and discuss the diagnostic and treatment challenges of this unusual case. This report, besides presenting a unique case, also highlights the important issues that need to be taken into account when assessing and managing patients with such complex conditions.

## Introduction

Recessive Dystrophic Epidermolysis Bullosa (RBED) is a rare and severe genetically determined disorder characterized by excessive susceptibility of the skin and mucosae to separate from the underlying tissues following mechanical trauma. Conversely, oligoarticular JIA is the commonest form of auto-immune arthritis in childhood. Both diseases are characterized by severe limitation of mobility and loss of function. Although the autoimmune bullous skin disease Epidermolysis Bullosa Aquisita (EBA) has been linked with other autoimmune conditions such as IBD, systemic lupus erythematosus, Hashimoto's thyroiditis and amyloidosis, there has not been any previous report of severe generalized RDEB associated with autoimmune conditions, such as arthritis.

## Case report

**Figure 1:**
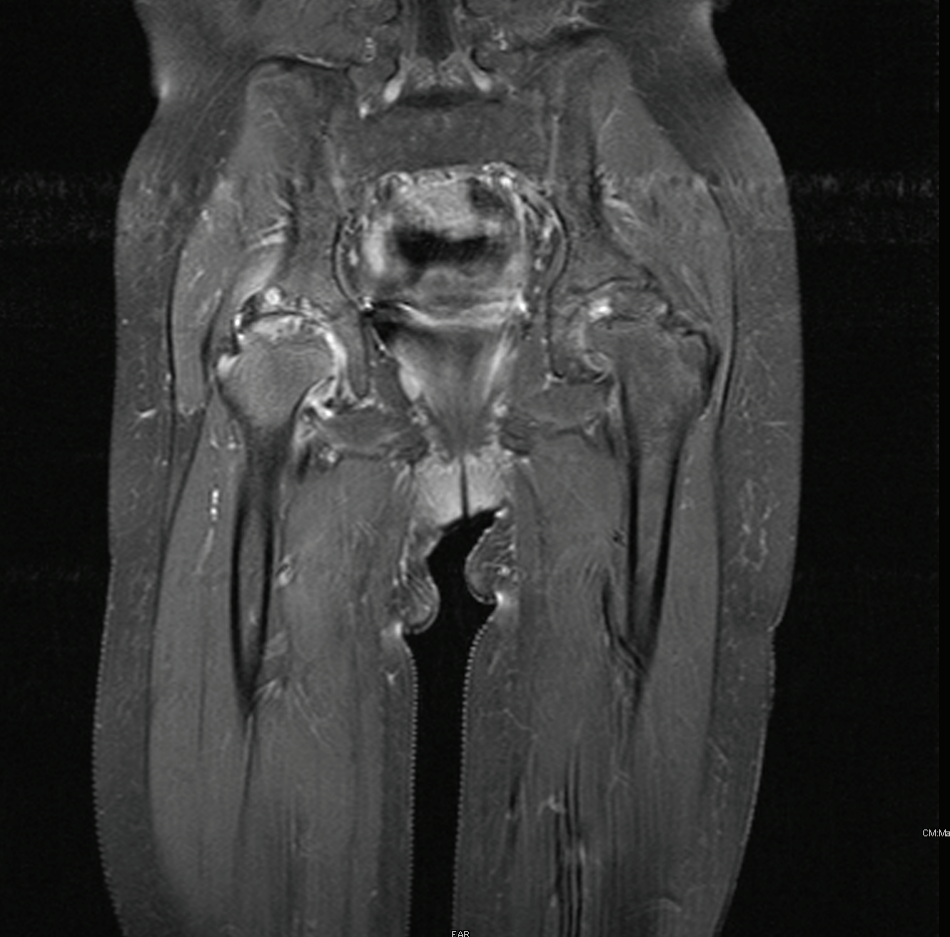
**MRI Scan of the Patient**. MRI with gadolinium showing active inflammation with synovial thickening, cortical irregularity, loss of cartilage and subcortical oedema on the right hip joint and chronic changes on the left hip joint.

Our patient was diagnosed with severe generalized RDEB shortly after birth on the basis of complete absence of type VII collagen and a sublamina densa split on electron microscopy of a skin biopsy. By the age of 4 years she developed abdominal distension and severe diarrhoea. Screen for celiac disease, food specific allergy tests (IgE's), and stool microscopy and culture were negative. An upper and lower gastrointestinal endoscopy and biopsy revealed the presence of an extensive yet non-classical colitis in keeping with unclassified inflammatory bowel disease. She was treated with a restrictive diet excluding dairy, wheat and soya and she was put on a modified feed. Sulfasalazine (1 gr/day) and low dose prednisolone (0.5 mg/kg/day) were added to try to control her bowel symptoms. Over time, chronic wounds due to her RDEB healed with atrophic scaring, and joint contractures developed despite regular physiotherapy, resulting in significant loss of mobility and function. At the age of 7 years she developed persistent localized pain in the right hip joint, associated with morning stiffness, antalgic position and rapid deterioration of function in that limb. Plain radiography of the hips showed erosive changes and loss of intra-articular joint space; MRI with gadolinium showed active inflammation with synovial thickening, cortical irregularity, loss of cartilage and subcortical oedema on the right and chronic changes on the left hip joint (Figure 1). She had mild microcytic anemia; ESR and CRP were moderately elevated. Anti-nuclear antibodies (ANA) titers were positive at 1:640 and she tested negative for HLA B-27. Screen for uveitis was negative. At this point this was considered a monoarthritis as the chronic changes seen on the left could be attributed to either previous disease activity or bio-mechanical causes; hence a synovial biopsy to exclude any other pathology would have been optimal. However, we faced a dilemma as whether to proceed with this invasive procedure in view of her extensive chronic skin erosions with high risk of infection as well as increased mortality risks of generalized anaesthesia in this group of patients [1,2]. The diagnosis of oligoarticular JIA was therefore based on clinical, laboratory and radiological findings. She was then treated with intra-articular joint injection (IAI) with steroids under USS guidance. Fluid aspirated was sterile for pathogens including mycobacterium tuberculosis.

A year following diagnosis, she presented with arthritis involving both knees and right hip; meanwhile her bowel symptoms were incompletely controlled; nutrition was suboptimal (weight: < 5^th ^centile, height: 10^th ^centile), and she required intensive dietitian support.

Her arthritis was treated with IAI with triamcinolone hexacetonide; she was started on oral methotrexate (15 mg/m^2^/week) that failed to control her disease. In view of the partially controlled colitis and progressive arthritis we decided to commence anti-TNF-α treatment. Adalimumab (20 mg/fortnightly) and methotrexate (15 mg/m^2^/week) both administered subcutaneously were chosen since the risk of sepsis precluded a permanent line and venous access was extremely difficult.

The combination of adalimumab and methotrexate has controlled both her arthritis and inflammatory bowel disease and she remained symptom free two years following initiation of treatment. In addition there was also a clinical improvement of her skin, with fewer chronically eroded areas and less marked chronic anaemia. Inflammatory markers and ANA titers improved. She subsequently developed two flares of her arthritis with no associated bowel symptoms, which was treated with IAI followed by physiotherapy and rehabilitation. Her nutritional status is satisfactory (weight and height: > 10^th ^centile).

## Discussion

Epidermolysis bullosa can broadly be separated into 4 major types and numerous subtypes according to the plane of blistering within the skin, in addition to the pattern of involvement and clinical severity. Severe generalized RDEB (previously known as Hallopeau-Siemens) is one of the most severe forms with an overall mortality rate of 8% during childhood [2]. The most common reported causes of death in childhood include sepsis and severe failure to thrive, while in the adolescent and adult population cutaneous squamous cell carcinoma accounts for the substantial majority of deaths, usually in the third to fifth decades. Other less common causes of death include renal failure and cardiomyopathy. Multidisciplinary care with early treatment of infections, optimizing nutrition and a wide variety of supporting services has improved the quality of life and survival rates over the past decade [3].

Nevertheless, clinical assessment and management of these patients remains challenging, especially as in our case additional nosological entities complicate the course of their illness. The presentation of persistent hip pain in a previously healthy child can pose a diagnostic dilemma with oligoarticular JIA being one of the possible diagnoses. In our patient there was a delay from initial onset of symptoms to establishing the diagnosis. Pain is also inevitable with this severe type of EB coupled with limitation of mobility from contractions and skin wounds. Additionally, the treatment with prednisolone and sulfasalazine for her IBD and regular use of NSAID for analgesia may have masked a more striking presentation. IBD arthropathy could be the cause of her joint symptoms; however the persistent oligoarticular pattern of presentation, the destructive form of arthritis, and the high ANA titers pointed towards a distinct diagnosis of JIA. Additionally, joint flare-ups persisted while bowel disease was in remission [4].

The routine approach to any patient presenting with monoarthritis of the hip would include synovial fluid aspiration +/- synovial biopsy. In this case the increased risk of secondary septic arthritis or sepsis in general precluded any intervention other than a therapeutic one; diagnosis was based on clinical and radiological findings alone.

With regards to treatment, although our patient had a common form of JIA, oral methotrexate failed to control her arthritis either because of innate disease resistance or inadequate gastrointestinal absorption. Oral instead of subcutaneous route was chosen based on family's preferences.

Introduction of anti-TNF-α biologics has altered the therapeutic approach to inflammatory conditions, over the last decade. The use of infliximab, a chimeric partly humanized monoclonal antibody, is well established in the treatment of both IBD and JIA in children. Placing a permanent intravenous catheter in a patient with DEB is very likely to have detrimental results given the high risk of secondary infection and sepsis. Adalimumab, a fully humanized IgG1 anti-TNF-α monoclonal antibody, is an approved drug for the treatment of JIA. Its use in IBD in adults is well documented and it has been recently reported as a safe and effective treatment for children with Crohn's disease [5]. In our case, it was considered as a safe alternative given its benefit for subcutaneous administration.

The patient remains asymptomatic for over 12 months and both her IBD and arthritis are well controlled. Nevertheless, immunological long-term complications of anti-TNF-α biologics are yet to be determined. Additionally, the long term risk of developing skin cancer reported in adults on anti-TNF treatment needs to be kept in mind especially in this case since patients with RDEB are more prone to epithelial squamous cell carcinomas; hence closed surveillance with regular skin examination is required.

The occurrence in our patient of RDEB, IBD and JIA is difficult to explain. Acquired EB has been linked with autoimmune conditions such as IBD, rheumatoid arthritis, and Hashimoto's thyroiditis with the pathophysiological mechanism involving production of IgG antibodies against an epitope of collagen type VII, the main component of anchoring fibrils at the dermo-epidermal junction (DEJ), and the mutated protein in dystrophic forms of EB [6]. Even in the absence of collagen VII in the joints, molecular mimicry might explain why these patients develop autoimmune arthritis. Similarly, in our case, it may be that fragility at the DEJ in RDEB leads to exposure of normally hidden autoantigens that subsequently trigger an immune reaction in other tissues including the joints.

## Conclusion

We have presented the unique case of a child with RDEB who also developed IBD and persistent oligoarticular JIA and discussed the important issues that need to be taken into account when assessing and managing patients with such complex conditions. Every step has to be cautious and constantly balanced against the risks associated with the intervention, as well as the long-term effects on the patient's quality of life.

## Key points

1. Children with rare diseases are similarly prone to develop other commoner conditions compared to the general population; therefore the newly presented persistent symptoms should be cautiously reviewed.

2. Treatment of patients with complex medical needs has to be guided primarily by the impact on the patient's quality of life as well as how will the new treatment affect or improve their life expectancy.

3. Multi-disciplinary team approach and effective communication of treatment options with the patient and family, facilitates decision making and improves the outcome.

## Consent

Written informed consent was obtained from the patient for publication of this case report and accompanying images. A copy of the written consent is available for review by the Editor-in-Chief of this journal.

## Competing interests

The authors declare that they have no competing interests.

## Authors' contributions

DM and DE were involved in the patient's care and prepared the manuscript. AM and JM were involved in the patient's care and reviewed the manuscript for its intellectual content. CP critically reviewed the manuscript. All authors read and approved the final manuscript.

## References

[B1] Culpepper TL Anaesthetic implications in epidermolysis bullosa dystrophica AANA J 2001 69 114 8 11759144

[B2] Fine JD Johnson LB Weiner M Suchindran C Cause-specific risks of childhood death in inherited epidermolysis bullosa J Pediatr 2008 152 276 80 10.1016/j.jpeds.2007.06.039 18206702

[B3] Atherton DJ Mellerio JE Denyer Harper JI, Oranje A, Prose N Epidermolysis bullosa Textbook of Paediatric Dermatology 2006 II, chapter 16 Blackwell Publishing 1291 1304

[B4] Caprilli R European evidence based consensus on the diagnosis and management of Crohn's disease: special situations Gut 2006 55 Suppl I i36 i58 10.1136/gut.2005.081950c PMC1859996 16481630

[B5] Rosh JR Retrospective Evaluation of the Safety and Effect of Adalimumab Therapy (RESEAT) in Pediatric Crohn's Disease Am J Gastroenterol 2009 104 3042 9 10.1038/ajg.2009.493 19724267

[B6] Remington J Chen M Burnett J Woodley DT Autoimmunity to type VII collagen: epidermolysis bullosa acquisita Curr Dir Autoimmun 2008 10 195 205 18460887 10.1159/000131455

